# Scribbled Optimizes BMP Signaling through Its Receptor Internalization to the Rab5 Endosome and Promote Robust Epithelial Morphogenesis

**DOI:** 10.1371/journal.pgen.1006424

**Published:** 2016-11-04

**Authors:** Jinghua Gui, Yunxian Huang, Osamu Shimmi

**Affiliations:** Institute of Biotechnology, University of Helsinki, Helsinki, Finland; Harvard Medical School, Howard Hughes Medical Institute, UNITED STATES

## Abstract

Epithelial cells are characterized by apical-basal polarity. Intrinsic factors underlying apical-basal polarity are crucial for tissue homeostasis and have often been identified to be tumor suppressors. Patterning and differentiation of epithelia are key processes of epithelial morphogenesis and are frequently regulated by highly conserved extrinsic factors. However, due to the complexity of morphogenesis, the mechanisms of precise interpretation of signal transduction as well as spatiotemporal control of extrinsic cues during dynamic morphogenesis remain poorly understood. Wing posterior crossvein (PCV) formation in *Drosophila* serves as a unique model to address how epithelial morphogenesis is regulated by secreted growth factors. Decapentaplegic (Dpp), a conserved bone morphogenetic protein (BMP)-type ligand, is directionally trafficked from longitudinal veins (LVs) into the PCV region for patterning and differentiation. Our data reveal that the basolateral determinant Scribbled (Scrib) is required for PCV formation through optimizing BMP signaling. Scrib regulates BMP-type I receptor Thickveins (Tkv) localization at the basolateral region of PCV cells and subsequently facilitates Tkv internalization to Rab5 endosomes, where Tkv is active. BMP signaling also up-regulates *scrib* transcription in the pupal wing to form a positive feedback loop. Our data reveal a unique mechanism in which intrinsic polarity genes and extrinsic cues are coupled to promote robust morphogenesis.

## Author Summary

Epithelial morphogenesis is one of the key processes in animal development. Evolutionarily conserved growth factors frequently instruct patterning and differentiation in morphogenesis. However, little is known about how extracellular cues and epithelial morphogenesis are mutually coordinated in vivo. Wing posterior crossvein (PCV) development in *Drosophila* provides an excellent system for understanding how bone morphogenetic protein (BMP) signaling regulates patterning and differentiation of epithelia. We find that the apical-basal polarity gene Scribbled (Scrib) is required for PCV formation by optimizing BMP signaling in the PCV region as follows. First, Scrib regulates BMP type-I receptor Thickveins (Tkv) localization basally. Second, Scrib facilitates Tkv internalization to the Rab5 endosomes to optimize signal transduction after receptor-ligand binding. Third, BMP signaling up-regulates *scrib* transcription in the pupal wing to form a positive feedback loop. These results suggest that coupling between epithelial polarity genes and conserved growth factors play crucial roles in patterning and differentiation of epithelia.

## Introduction

Epithelial cells are characteristically polarized with apical-basal plasma membrane polarity, which is crucial for tissue homeostasis [1,2]. Many of intrinsic factors underlying apical-basal polarity have been identified to be tumour suppressors [1,2]. Multiple distinct but interacting groups of proteins, including the partitioning defective (PAR) proteins, the Crumb (Crb) complex and the Scribbled (Scrib) complex, play crucial roles in regulating epithelial polarity [1]. Among these, the Scrib complex, composed of Scrib, Discs large 1 (DLG1) and Lethal giant larvae (LGL) [3], has previously been identified as a basolateral determinant of epithelial cells by instructing epithelial-specific cytoskeletal rearrangements, the distribution of apical proteins, and polarized trafficking machinery [3–5]. Strong loss-of-function (LOF) of the Scrib complex causes tumor-like growths [4,5].

Decapentaplegic (Dpp), a conserved *Drosophila* bone morphogenetic protein (BMP)-type ligand, plays crucial roles in various developmental contexts [6]. The Dpp ligands bind to the type I receptor Thickveins (Tkv) and type II receptor Punt. This stimulates Tkv to phosphorylate the transcriptional factor Mad. Upon phosphorylation, Mad binds co-Smad Medea and then translocates to the nucleus for transcriptional regulation of target genes. During pupal stages, Dpp is produced in the longitudinal veins (LVs) of the pupal wing and trafficked into the posterior crossvein (PCV) region from around 18 hr after pupariation (AP) to establish long-range signaling [7]. Prior to *dpp* transcription in the PCV region at around 26 hr AP [8], long-range BMP/Dpp signaling appears to instruct PCV patterning and differentiation, since either loss- or gain-of-function of Dpp trafficking is sufficient for causing crossveinless or ectopic crossvein phenotypes [9,10]. Dpp trafficking is supported by the BMP binding proteins short-gastrulation (Sog) and crossveinless (Cv) to direct Dpp ligand localization in the PCV region [7]. Since the Dpp trafficking mechanism appears to limit ligand available in the PCV region [7,9], systems that optimize the signaling response to limiting external ligands must be crucial. Thus PCV formation serves as a unique model to address molecular mechanisms underlying the optimization of BMP signaling [6,11–15].

Epithelial morphogenesis is one of the key processes in animal development and is frequently regulated by highly conserved signaling molecules. To promote robust morphogenesis, ensuring precise interpretation of signal transduction as well as spatiotemporal distribution of extracellular ligands are important. Here, using PCV formation in *Drosophila* as a model, we demonstrate that the basolateral determinant Scrib complex is required for PCV formation through optimizing BMP signaling. Scrib regulates Tkv localization by facilitating its meeting extracellular ligands, and subsequently is involved in Tkv internalization to Rab5 endosomes. Our data suggest a mechanism that optimizes the signaling response to limiting external ligands for patterning and differentiation of epithelia.

## Results

### Scrib is required for PCV formation

**Fig 1:**
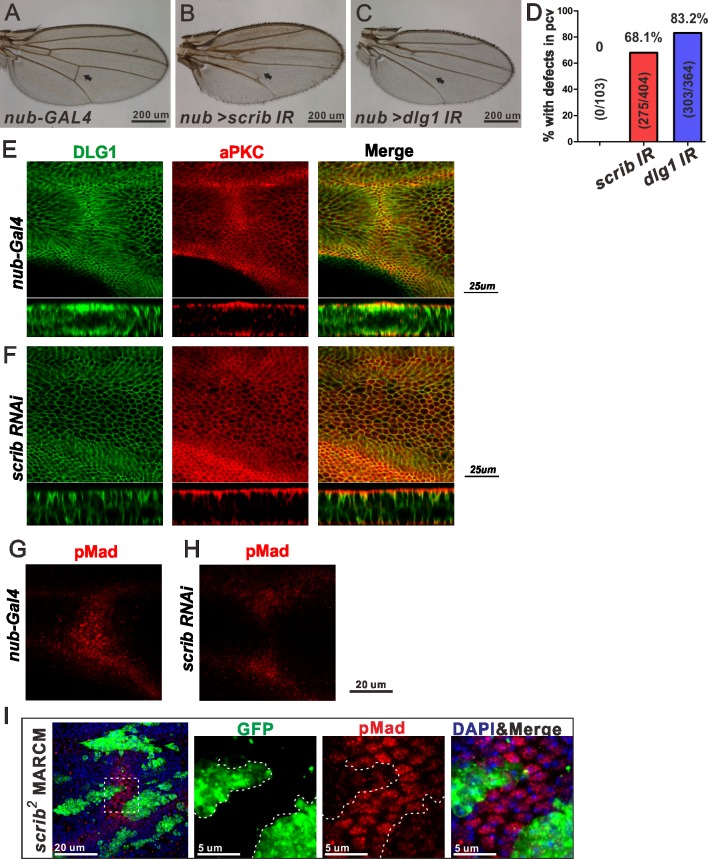
The Scrib complex is required for PCV formation through regulating BMP/Dpp signaling. (A-C) Adult wings of control (*nub-GAL4*, *GAL80*^*ts*^ *(nub*^*ts*^*)*) (A), *scrib* RNAi (*nub*^*ts*^ *> scrib* RNAi) (B) and *dlg1* RNAi (*nub*^*ts*^ *> dlg1* RNAi) (C). RNAi flies were cultured at 25 (A), 25 (B), and 27 (C) °C, respectively. The PCV regions are indicated by arrows. (D) Quantification of adult wing phenotypes in A-C. (E, F) Pupal wings at 24 h AP showing aPKC and DLG1 staining in control (*nub*^*ts*^) (E) and *scrib* RNAi (*nub*^*ts*^ *> scrib* RNAi) (F). Optical cross sections focused on the PCV region are shown at the lower panel. aPKC localizes apically and DLG1 basolaterally in both control and *scrib* RNAi wings. Note that the lumen of the wing vein is formed in control but not in *scrib* RNAi wings. (G, H) pMad staining in the PCV region in control (*nub*^*ts*^) (G) and *scrib* RNAi (*nub*^*ts*^ > *scrib* RNAi) (H) at 24 h AP. (I) Effects of *scrib* mutant clones on pMad (red) at 24 h AP in the PCV region. Mutant GFP-labeled cells (green) were generated using MARCM system. Dashed box in left panel depicts the region of interest (ROI). Higher magnification pictures of the ROI are shown in the right panels.

In a screen for novel components involved in PCV formation, we identified the components of the Scrib complex as candidates. To investigate how Scrib regulates PCV formation, we analyzed the Scrib complex in the *Drosophila* pupal wing using a conditional knockdown approach [16]. We observed that knockdown of *scrib*, *dlg1* or *lgl* in the pupal wing blade disrupted PCV formation (Fig 1A–1D and S1A Fig). Notably, knocking down Scrib complex components in this manner did not disrupt cell polarity, as shown by the apical marker aPKC and the basolateral marker DLG1 (Fig 1E and 1F). Thus a partial reduction in Scrib complex gene expression is sufficient to disrupt PCV morphogenesis but not to disrupt apical-basal polarity, indicating that loss of the Scrib complex disrupts PCV formation by another mechanism. To understand whether the PCV-less phenotypes resulted from reduced BMP signaling, phosphorylated Mad (pMad) staining (a readout of BMP signaling [17]) was used to examine the effects of *scrib* knock down in the PCV region. Knockdown of *scrib* in the wing caused a loss of BMP signaling in the PCV region (Fig 1G and 1H). *scrib* or *dlg1* mutant clone analysis further suggested that Scrib and DLG1 are required for BMP signaling in the PCV region in a cell-autonomous manner (Fig 1I and S1B Fig).

**Fig 2:**
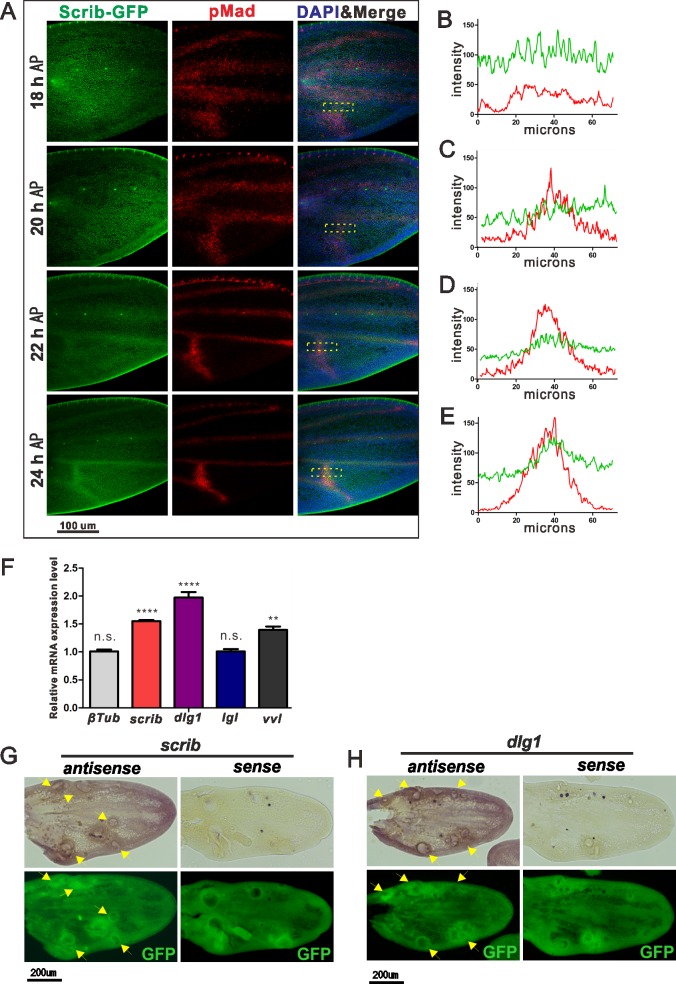
BMP signal regulates *scrib* transcription in the pupal wing. (A) Scrib-GFP (green), pMad staining (red), and DAPI (blue) at 18 to 24 h AP. Nuclei are marked by DAPI (blue) staining. (B-E) Plot profile analysis of Scrib-GFP (green) and pMad (red) in ROIs in A, corresponding to 18h AP, 20h AP, 22h AP and 24h AP, respectively. (F) Quantitative PCR analysis of mRNA levels in pupal wings at 24 h AP. mRNA levels in *dpp*^*shv*^ *> ca-tkv* flies were compared to control *yw* flies. Relative mRNA expression level of genes of interest was calculated with normalization to *gapdh* mRNA level. *ventral veinless* (*vvl*) was used as a positive control. Error bar, SEM. ****P<0.001, **P<0.01, two-paired *t*-test. (G, H) In situ hybridization analysis of *scrib* (G) and *dlg1* (H) in pupal wings at 24 h AP (upper panels). caTkv expression clones were generated using MARCM. Clones were marked by GFP (lower panels). Up-regulated *scrib* or *dlg1* corresponding to caTkv clones are indicated by arrows. Sense probe was applied to the wings expressing caTkv clones as a negative control.

Loss of BMP signal in *scrib* clones in the PCV region appears not be caused by perturbed ligand trafficking. GFP-Dpp ligands expressed in *scrib* clones are rather diffusible (S1C–S1F Fig). These results support that Scrib is required for optimizing BMP signal in the PCV region but not for facilitating BMP ligand trafficking. Interestingly, Scrib proteins are enriched in the vein primordial cells at 24 hr AP, where BMP signaling is positive (Fig 2A). These observations indicate that the Scrib complex may be up-regulated by BMP signaling and may constitute a feedback loop. To test this idea, we assessed pMad signaling and Scrib protein staining in pupal wings at different time points. At 18 h AP, the pMad signal is widely observed in the prospective LV regions, but faint in the PCV region, in line with previous reports that PCV morphogenesis initiates around 18h AP (Fig 2A) [7,8]. pMad staining becomes refined in LVs, and intensifies in the PCV around 20–24 hr AP (Fig 2A–2E). Scrib is uniformly distributed throughout wing tissues at 18 h AP, when BMP signaling is low. Scrib then gradually accumulates in the vein progenitor cells while BMP signaling becomes refined at 20–24 hr AP (Fig 2A–2E). Based on these data, we postulate that the BMP/Dpp signal may regulate *scrib* transcription.

To study whether transcriptional levels of *scrib* and *dlg1* are also susceptible to BMP/Dpp signal activity, we performed quantitative PCR (qPCR) and found that *scrib* and *dlg1* are transcriptionally regulated by BMP/DPP signaling (Fig 2F). Our data also indicate that *scrib* and *dlg1* transcription are up-regulated by BMP signal in future intervein cells as well as wing vein progenitor cells, when the constitutively active form of Tkv (caTkv) are ectopically induced (Fig 2G and 2H). To understand whether Scrib levels are up-regulated as vein material or specifically up-regulated by BMP signal, we compared Scrib and DE-cadherin (DE-Cad), a key molecule for epithelial morphogenesis [18], by producing ectopic clones of caTkv. Interestingly, up-regulation of Scrib but not DE-Cad become visible already at 20 h AP (S1G Fig). DE-Cad then becomes up-regulated at 24 h AP (S1H Fig), suggesting that both Scrib and DE-Cad are positively regulated by BMP signaling but in a distinct manner. Together, our data suggest that feedback through Scrib and DLG1 is crucial for long-range BMP/Dpp signaling during PCV development.

### Scrib regulates Tkv distribution in the PCV region

**Fig 3:**
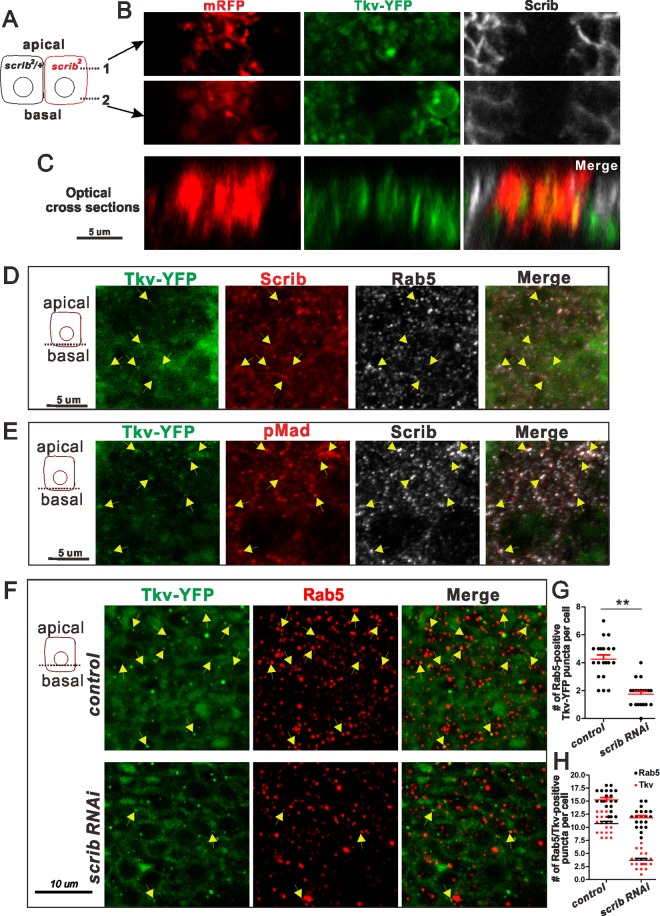
Scrib regulates spatial distribution of Tkv in the PCV region. (A) A schematic of different planes (1 and 2) of PCV cells along the apicobasal axis in B. (B) Loss of Scrib affects Tkv distribution. *scrib* mutant clones are marked by mRFP and absence of Scrib staining. Tkv-YFP staining in the PCV region at 24 h AP. (C) Optical cross sections of B. Note that Tkv is more enriched basally in control but restricted more apically in *scrib* mutant cells. (D, E) Wild-type pupal wing. Tkv-YFP, Scrib and Rab5 staining in the PCV region at 24 h AP (D). Tkv-YFP, pMad and Scrib staining in the PCV region at 24 h AP (E). Arrows indicate that Tkv-YFP puncta co-localize with Scrib and Rab5 (D), or pMad and Scrib (E). (F) Tkv-YFP and Rab5 staining at the basolateral plane in control (*nub*^*ts*^) and *scrib* RNAi (*nub*^*ts*^ *> scrib RNAi*) in the PCV region at 24 h AP. Arrows indicate that Tkv-YFP puncta co-localize with Rab5. (G) Quantification of co-localization of Tkv-YFP with Rab5. n = 20 cells for each sample. (H) Quantification of Tkv-YFP or Rab5 puncta. n = 20 cells for each sample. Error bar, SEM. **P<0.01 in f, two-paired *t*-test. Images are the composite of sections with 2 μm thickness in D-F. Images have been taken by a 25X objective for B, C, and 63X objective for D-F.

How does Scrib affect long-range BMP/Dpp signaling in the PCV region? During the course of Dpp trafficking into the PCV region, Dpp ligands appear to move through the basal side of the pupal wing epithelia [9]. Since the Scrib complex has been proposed to regulate the apical-basal protein distribution of epithelial cells [4,19], we hypothesize that Scrib may affect BMP receptor localization. To test this hypothesis, we investigated the distribution of the BMP type I receptor Tkv in the PCV region. In wild-type pupal wings, Tkv is located at the basolateral region of PCV cells, however Tkv localization was observed more apically in *scrib* mutants (Fig 3A–3C and S2A and S2B Fig). To investigate whether Tkv localization is regulated by Scrib or modulated as a secondary affect of changing cell polarity, we used an RNAi approach. Our data reveal that Tkv is enriched apically but reduced basally in *scrib* RNAi wings (S2C and S2D Fig). These results suggest that Scrib ensures enhanced basal Tkv localization where BMP ligand trafficking takes place. We also found that Tkv was frequently observed in puncta at the basal side of the PCV region (Fig 3D and 3E). We wondered whether the observed amounts of Tkv reflect internalized receptors. At the basal side of the PCV cells, Tkv often co-localizes with Scrib and DLG1 at the Rab5-positive early endosomes (Fig 3D and S2E Fig). A key process in canonical BMP signal transduction is production of pMad by activated receptors and its accumulation in the nucleus. In wild type cells, substantial amounts of pMad co-localized with Tkv and Scrib as puncta in early endosomes (Fig 3E). Since pMad subsequently accumulates in the nucleus, endosome-localized pMad appears to be transient. Our data also reveal that the number of co-localization of Tkv with Rab5 is decreased in *scrib* RNAi wings due to less number of Tkv puncta (Fig 3F–3H and S2F Fig). Taken together, these data suggest that Scrib-dependent Tkv localization at Rab5 endosomes is key for producing robust BMP signaling in the PCV region.

### Clathrin-dependent endocytosis is important for BMP signaling in the PCV region

**Fig 4:**
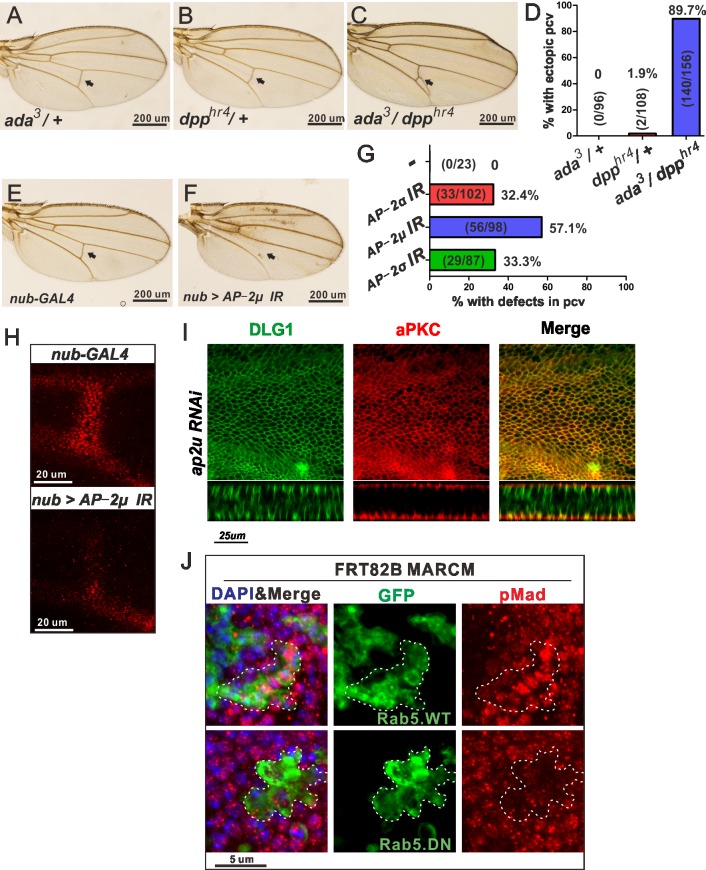
Clathrin dependent endocytosis is required for BMP/Dpp signaling during PCV formation. (A-C) Adult wings of *ada*^*3*^*/+* (A), *dpp*^*hr4*^*/+* (B) and *ada*^*3*^*/dpp*^*hr4*^ (C). The PCV regions are indicated by arrows. (D) Quantification of adult wing phenotypes in A-C. (E, F) Adult wings of control (*nub*^*ts*^) (E) and *AP-2u* RNAi *(nub*^*ts*^ *> AP-2u* RNAi) (F). The PCV regions are indicated by arrows. (G) Quantification of adult wing phenotypes in E, F and S3A and S3B Fig. (H) pMad staining in the PCV region at 24 h AP in control (upper panel) or *AP-2u* RNAi (lower panel). (I) Pupal wing at 24 h AP showing aPKC and DLG1 staining in *AP-2u* RNAi RNAi *(nub*^*ts*^ *> AP-2u* RNAi). Optical cross section focused on the PCV region (lower panels). (J) Effects of clonal overexpression of Rab5.WT (upper panel) or Rab5.DN (lower panel) on pMad staining (red) in the PCV region at 24 h AP. Rab5-expressing clones are marked by GFP (green). Nuclei are marked by DAPI (blue) staining. Dashed lines depict overexpression clones.

If Tkv localization at Rab5 endosomes is key for BMP signaling in the PCV region, ablation of receptor internalization may reduce the BMP signal. Speculating that Scrib facilitates Tkv internalization through a clathrin-dependent mechanism, we investigated whether clathrin-dependent endocytosis affects BMP signaling in the PCV region. Adaptor complex-2 (AP-2), which functions together with clathrin to initiate endocytosis in the plasma membrane, is composed of four subunits: *α*, *β*, *μ* and *σ* [20]. Due to its importance in various cellular functions, complete inhibition of endocytosis causes severe tissue damage. Therefore, the relevance of endocytosis was analyzed by investigating genetic interactions and weak loss-of-function analysis. First, we analyzed genetic interactions of *dpp* alleles with *AP-2α (ada)*, and found that *ada* interacted genetically with *dpp* in the PCV region (Fig 4A–4D). Ectopic PCV phenotypes are occasionally observed when Dpp signaling in the PCV region is partially disturbed [21]. This might be caused by incomplete maintenance of positive feedback mechanisms, resulting in ectopic ligand trafficking. Next we found that RNAi of AP2 complex subunits shows crossveinless phenotypes (Fig 4E–4G and S3A and S3B Fig). The PCV-less phenotypes were confirmed to be due to loss of BMP signaling in the PCV region, since the pMad signal was largely ablated in the prospective PCV region of wings expressing *AP-2μ* RNAi (Fig 4H). Importantly, knocking down *AP-2μ* in this condition did not disrupt epithelial polarity, as shown by the apical localization of aPKC and the basolateral localization of DLG1 (Fig 4I). We then investigated whether the early endosome protein Rab5 is required for BMP signaling in the PCV region. Ectopic expression of a dominant negative form of Rab5 significantly reduced BMP signaling in the PCV region (Fig 4J). These results suggest that clathrin-mediated internalization of the BMP receptor to Rab5 endosomes is a crucial step for BMP signaling in the PCV region.

### Scrib interacts with Tkv

**Fig 5:**
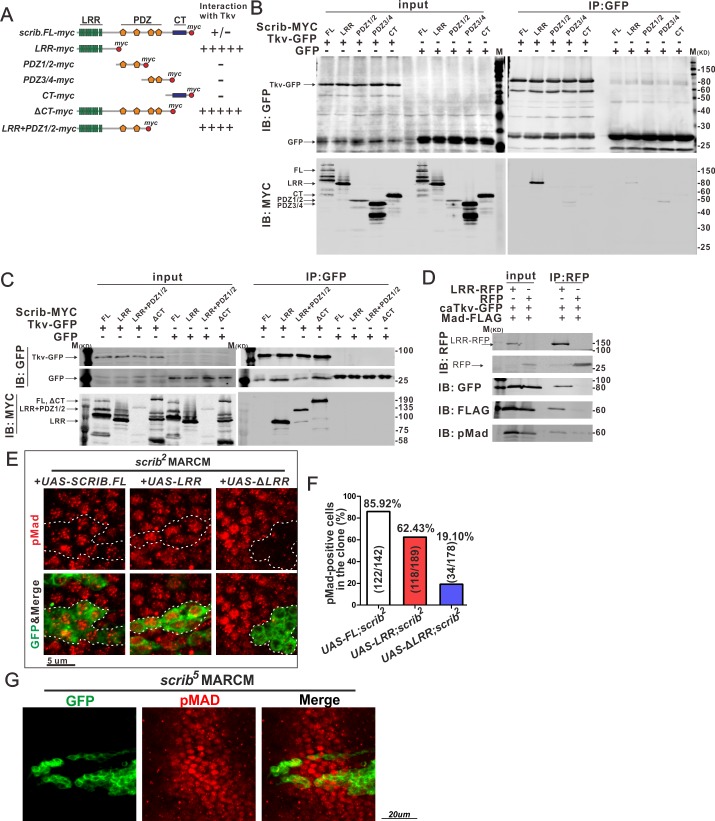
LRR domain of Scrib interacts with Tkv. (A) Diagram of different fragments of Scrib. Intensities of interaction between Scrib fragments and Tkv are shown at the right. (B, C) Co-IP of Scrib (full length or fragments) and Tkv. Scrib-MYC and Tkv-GFP were expressed in S2 cells, and cell lysates were immunopreciated by anti-GFP. Cell lysates (input) and immunoprecipitated proteins (IP: GFP) were analysed by Western blots probed with anti-GFP and anti-MYC antibodies. (D) Co-IP of LRR, Tkv and Mad. LRR-RFP, caTkv-GFP and Mad-FLAG were expressed in S2 cells, and cell lysates were immunopreciated by anti-RFP. Cell lysates (input) and immunoprecipitated proteins (IP: RFP) were analysed by Western blots probed with anti-GFP, anti-FLAG and anti-pMad antibodies. (E) Expression of Scrib (full length), LRR domain or Scrib without LRR domain in *scrib* mutant clones labeled by GFP. pMad staining in PCV region at 24 h AP. Dashed lines delineate the clone boundaries. (F) Quantification of pMad-positive cells in E. (G) Effects of *scrib*^*5*^ mutant clones on pMad (red) at 24 h AP in the PCV region. Mutant GFP-labeled cells (green) were generated using MARCM. Results shown are representative of one of three independent experiments (B-D).

How then does Scrib play a role in Tkv internalization and signaling? First, we addressed whether physical interactions between Scrib and Tkv are key for Scrib function. Scrib belongs to the LAP protein family, containing Leucine-rich repeats (LRR), PDZ and C-terminal (CT) domains (Fig 5A). To investigate whether Tkv and Scrib are physically associated, we performed co-immunoprecipitation (co-IP) analysis. GFP-tagged Tkv and MYC-tagged full-length or truncated Scrib were co-transfected into *Drosophila* S2 cells. Our data suggest that full-length Scrib weakly associates with Tkv (S4A Fig). In contrast, when different domains of Scrib were co-expressed, LRR regions showed strong interactions with Tkv (Fig 5B), suggesting that the LRR domain is sufficient for interaction with Tkv. Previous studies suggest that the LRR domain is mainly responsible for cell polarity maintenance and proliferation control, while the PDZ domain mediates physical interactions with a variety of proteins [22]. A Scrib fragment including the LRR and PDZ domains, but not the CT domain, showed strong interactions with Tkv (Fig 5A and 5C). Since the CT domain is capable of binding to LRR (S4B Fig), this domain may function as a regulator of Scrib-Tkv interactions. Next, we examined whether the conformational change conferred by activation affects the association of Tkv with Scrib. Although caTkv associates with full-length Scrib less efficiently (S4A Fig), LRR interacts with caTkv and pMad (Fig 5D), suggesting that Tkv complexed with Scrib is able to phosphorylate Mad. We then tested whether LRR is sufficient for maintaining BMP signaling in the PCV region in vivo. BMP signaling in *scrib* mutant clones in the PCV region was sufficiently restored by LRR as well as by full-length Scrib, but not by Scrib without an LRR domain (Fig 5E and 5F). Consistently, pMad signaling in the PCV region remains intact in mutant cells of *scrib*^*5*^ allele that lacks the PDZ3/4 and CT domains (Fig 5G) [22]. We further confirmed that the LRR, but not the PDZ domain substantially restores cell polarity in *scrib* mutant cells of the pupal wings (S4C Fig). Therefore, restoration of BMP signaling in *scrib* mutant clones LRR might be mediated through biochemical interaction of Scrib, Tkv and pMad in endosomes or through restoration of tissue polarity. However, our RNAi data in which Scrib regulates BMP signaling in the PCV region in a distinct manner from regulation of tissue polarity (Fig 1) sufficiently support that the former scenario is more likely. Taken together, these results indicate that the LRR domain not only sustains epithelial polarity, but also interacts with the BMP receptor to maintain BMP signaling.

### Rab5 and Scrib form a complex with Tkv

**Fig 6:**
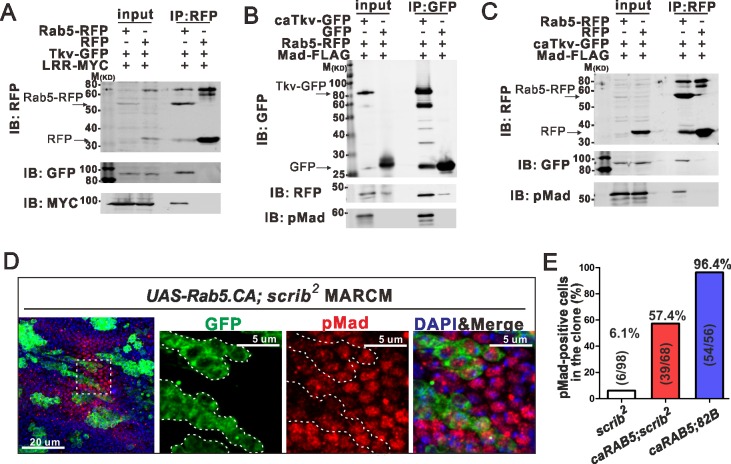
Rab5 and LRR domain of Scrib form a complex with Tkv. (A) Co-IP of Rab5, Tkv and LRR domain of Scrib. Rab5-RFP, Tkv-GFP and LRR-MYC were expressed in S2 cells, and cell lysates were immunoprecipitated by anti-RFP. Cell lysates (input) and immunoprecipitated proteins (IP-RFP) were analyzed by Western blot probed with anti-RFP, anti-GFP and anti-MYC antibodies. (B, C) Co-IP of Rab5, caTkv and Mad. Rab5-RFP, caTkv-GFP and Mad-FLAG were expressed in S2 cells, and cell lysates were immunoprecipitated by anti-GFP or anti-RFP. Cell lysates (input) and immunoprecipitated proteins (IP: GFP in B, or IP: RFP in C) were analyzed by Western blot probed with anti-RFP, anti-GFP and anti-pMad antibodies. Results are representative of one of three independent experiments (A-C). (D) Expression of constitutively active Rab5 in *scrib* mutant clones labeled by GFP. pMad staining in PCV region at 24 h AP. Dashed lines delineate the clone boundaries. Nuclei are marked by DAPI (blue) staining. (E) Quantification of pMad positive cells in *scrib* mutant cells of D. Results are collected from 3 independent experiments.

How is internalized Tkv regulated to effect BMP signaling? One simple hypothesis is that Tkv preferentially localizes to Rab5 endosomes to optimize BMP signaling. In that case, Tkv may associate with Rab5 as well as with Scrib to form a complex in the early endosome. To test this hypothesis, Tkv, Rab5 and LRR were co-expressed in S2 cells. We found that Tkv, Rab5 and LRR form a protein complex (Fig 6A). When caTkv and Rab5 are co-expressed, they not only interact with each other, but also with pMad (Fig 6B and 6C and S5A Fig), suggesting that Tkv in the Rab5 endosome is capable of phosphorylating Mad. Tkv is able to associate with various forms of Rab5, including wild-type, constitutively active (Q88L) and dominant negative (S43N) (S5B Fig) [23]. Furthermore, the BMP type-II receptor Punt was also detected in the complex with Rab5 and LRR (S5C Fig). These results are consistent with our hypothesis that BMP receptors are preferentially localized in the Rab5 endosome to optimize BMP signaling. We further found that the constitutively active form of Rab5 partially rescued loss of BMP signaling in *scrib* mutants (Fig 6D and 6E), suggesting that the Rab5 endosomes play significant roles in BMP signaling during PCV formation.

## Discussion

This study shows that the Scrib complex, a basolateral determinant, is a novel feedback component that optimizes BMP signaling in the PCV region of the *Drosophila* pupal wing.

During PCV development, limited amounts of Dpp ligands are provided by the Dpp trafficking mechanism [7,9]. Furthermore, amounts of receptors appear to be limited since *tkv* transcription is down-regulated in the cells in which the BMP signal is positive [7,24], a mechanism that serves to facilitate ligand diffusion and sustain long-range signaling in the larval wing imaginal disc [25,26]. To provide robust signal under conditions in which both ligands and receptors are limiting, additional molecular mechanisms are needed. Previous studies suggest that two molecules play such roles. Crossveinless-2 (Cv-2), which is highly expressed in the PCV region, serves to promote BMP signaling through facilitating receptor-ligand binding [12,27]. Additionally, the RhoGAP protein Crossveinless-c (Cv-c) provides an optimal extracellular environment to maintain ligand trafficking from LVs into PCV through down-regulating integrin distribution at the basal side of epithelia [9]. Importantly, both *cv-2* and *cv-c* are transcriptionally regulated by BMP signaling to form a feedback or feed-forward loop for PCV formation.

Scrib, a third component, sustains BMP signaling in the PCV region in a different manner. First, to utilize Tkv efficiently, Scrib regulates Tkv localization at the basolateral region in the PCV cells, where ligand trafficking takes place. Regulation of receptor localization could be a means of spatiotemporal regulation of signaling molecules during the dynamic process of morphogenesis. Second, to optimize the signal transduction after receptor-ligand binding, Scrib facilitates Tkv localization in the Rab5 endosomes. Localization of internalized Tkv is abundant at Rab5 endosomes in the PCV region of wild-type, but not *scrib* RNAi cells. While the physical interaction between Scrib, Tkv and Rab5 in the pupal wing remains to be addressed, our data in S2 cells suggest that physical interactions between these proteins are key for preferential localization of Tkv at the Rab5 endosomes. Recently, Scrib has been implicated in regulating NMDA receptor localization through an internalization-recycling pathway to sustain neural activity [28]. Therefore, Scrib may be involved in receptor trafficking in a context-specific manner, the molecular mechanisms of which, however, remain to be characterized. Third, BMP/Dpp signaling up-regulates *scrib* transcription in the pupal wing. Moreover, knockdown of *scrib* leads to loss of BMP signaling in PCV region but not loss of apical-basal polarity (Fig 1). These facts suggest that upregulation of Scrib is key for optimizing BMP signaling by forming a positive feedback loop.

Previous studies indicate that cell competition takes place between *scrib* clones and the surrounding wild-type tissues in the larval wing imaginal disc [29]. Moreover, cell competition has been documented between loss-of-Dpp signal and the surrounding wild-type tissues [30]. We presume that the mechanisms proposed in this study are independent of cell competition for the following reasons. First, *scrib* RNAi and *AP-2μ* RNAi data reveal that loss of BMP signal in the PCV region is produced without affecting cell polarity (Figs 1 and 3). Thus, cell competition is unlikely to occur in this context. Second, BMP signal is intact in *scrib* mutant clones of the wing imaginal disc (S6 Fig), suggesting that cell competition caused by *scrib* clones is not a direct cause of loss of BMP signaling in *scrib* mutant cells.

Previous studies established that receptor trafficking plays crucial roles in signal transduction of conserved growth factors, including BMP signaling. Several co-factors have been identified as regulators of BMP receptor trafficking [31–38]. Some of them down-regulate BMP signaling [31–35,38], while others help maintain it [36,37]. We propose that the Scrib-Rab5 system is a flexible module for receptor trafficking and can be utilized for optimizing a signal. During larval wing imaginal disc development, BMP ligands are trafficked through extracellular spaces to form a morphogen gradient. Although previous studies indicate that regulation of receptor trafficking impacts BMP signaling in wing imaginal discs [23,39], BMP signaling persists in *scrib* or *dlg1* mutant cells in wing discs (S6 Fig). Wing disc cells interpret signaling intensities of a morphogen gradient. In this developmental context, an optimizing mechanism might not be beneficial to the system. In contrast, cells in the PCV region use the system to ensure robust BMP signaling.

Taken together, our data reveal that a feedback loop through BMP and Scrib promotes Rab5 endosome-based BMP/Dpp signaling during PCV morphogenesis. Since the components (BMP signaling, the Scrib complex, and Rab5 endosomes) discussed in this work are highly conserved, similar mechanisms may be widely utilized throughout Animalia.

## Materials and Methods

### Fly strains and genetics

UAS-*scrib* RNAi (#35748), *scrib*^*2*^ (#41755), UAS-*dlg1* RNAi (#35772), *dlg1*^*B*^ *FRT19A*/FM7 (#57087), UAS-*lgl* RNAi (#35773), UAS-*AP-2μ* RNAi (#28040), UAS-*AP-2α* RNAi (#32866), UAS-*AP-2σ* RNAi (#27322), UAS-*tkv*^*Q253D*^ (#36536), UAS-*myrRFP* (#7118), UASp-*Rab5*^*Q88L*^*-YFP* (#9774), UASp-*Rab5*^*S43N*^*-YFP* (#9771), UAS-*scrib* (#59079), UAS-*scrib*.*LRR*.*LASPD* (#59081) and UAS-*scrib*.*DeltaLRR*.*GFP* (#59084), *nubbin*-Gal4 (*nub-GAL4*) (#25754) and *tub-GAL80*^*ts*^ (#7018) were obtained from the Bloomington *Drosophila* Stock Center. Scrib-GFP (#CA07683) was obtained from Fly Trap projects [40,41]. Tkv-YFP (#115298) was obtained from the Kyoto *Drosophila* Genetic Resource Center. *dpp*^*hr4*^, *dpp*^*shv*^-Gal4, UAS-*tkv-HA*, UAS-*GFP-dpp* were described previously [7,9]. *UAS-myr-deleteLRR and Scrib*^*5*^ were obtained from D. Bilder, *ada*^*3*^ from M. Gonzalez-Gaitan [42]. Fly stocks were maintained at 25°C unless otherwise mentioned. To induce MARCM clones [43], larvae were heat-shocked for two hours at 37°C at 96 hours after egg laying (AEL).

### Full Genotypes

Fig 1A, 1E and 1G: *w/+; nub-Gal4/+; Tub-Gal80*^*ts*^*/+*; B, F and H: *w/+; nub-Gal4/+; Tub-Gal80*^*ts*^*/scrib RNAi*; C: *w/+; nub-Gal4/+; Tub-Gal80*^*ts*^*/ dlg1 RNAi*; I: *hsFlp/+; tub-Gal4*, *UAS-mCD8-GFP/+; FRT82B tub-Gal80*/*FRT82B scrib*^*2*^

Fig 2A–2E: *w;; Scrib-GFP*; F: *yw* as a control*; w+; UAS-tkv*.*Q253D/+; dpp*^*shv*^*-Gal4/+*; G, H: *hsFlp/+; tub-Gal4*, *UAS-mCD8-GFP/ UAS-tkv*.*Q253D; FRT82B*, *tub-Gal80*/*FRT82B*

Fig 3B and 3C: *hsFlp/+; UAS-myr-mRFP/Tkv-YFP; tub-Gal4 FRT82B tub-Gal80*/*FRT82B scrib*^*2*^; D-E: *w; Tkv-YFP/+*; F: *w/+; nub-Gal4/Tkv-YFP; Tub-Gal80*^*ts*^*/+; w/+; nub-Gal4/Tkv-YFP; Tub-Gal80*^*ts*^*/scrib RNAi*

Fig 4A: *yw; ada*^*3*^*/+*; B: *yw; dpp*^*hr4*^*/+*; C: *yw; dpp*^*hr4*^*/ ada*^*3*^; E and H: *w/+; nub-Gal4/+; Tub-Gal80*^*ts*^*/ +*; F, H and I: *w/+; nub-Gal4/+; Tub-Gal80*^*ts*^*/ AP-2μ RNAi*; J: *hsFlp/+; tub-Gal4*, *UAS-mCD8-GFP/* UASp-*RAB5*^*WT or S43N*^*-YFP; FRT82B*, *tub-Gal80*/*FRT82B*

Fig 5E and 5F: *hsFlp/+; tub-Gal4*, *UAS-mCD8-GFP/UAS-Scrib*.*fl-GFP(or UAS-scrib*.*LRR*.*LASPD or UAS-scrib*.*DeltaLRR*.*GFP); FRT82B tub-Gal80*/*FRT82B scrib*^*2*^; G: *hsFlp/+; tub-Gal4*, *UAS-mCD8-GFP/+; FRT82B tub-Gal80*/*FRT82B scrib*^*5*^

Fig 6D: *hsFlp/+; tub-Gal4*, *UAS-mCD8-GFP/ UASp-RAB5*^*Q88L*^*-YFP; FRT82B tub-Gal80*/*FRT82B scrib*^*2*^

S1A Fig: *w/+; nub-Gal4/+; Tub-Gal80*^*ts*^*/lgl RNAi*;

B: *FRT19A dlg1*^*B*^*/ hsFlp*, *tub-GAL80*, *FRT19A; tub-GAL4*, *UAS-mCD8-GFP/+*; C and E: *hsFlp/+; UAS-myr-mRFP/UAS-GFP-Dpp; tub-Gal4 FRT82B tub-Gal80*/*FRT82B*; D and F: *hsFlp/+; UAS-myr-mRFP/UAS-GFP-Dpp; tub-Gal4 FRT82B tub-Gal80*/*FRT82B scrib*^*2*^; G, H: *hsFlp/+; tub-Gal4*, *UAS-mCD8-GFP/ UAS-tkv*.*Q253D; FRT82B*, *tub-Gal80*/*FRT82B*

S2B Fig: *hsFlp/+; UAS-myr-mRFP/Tkv-YFP; tub-Gal4 FRT82B tub-Gal80*/*FRT82B scrib*^*2*^; C: *w/+; nub-Gal4/Tkv-YFP; Tub-Gal80*^*ts*^*/+*; D: *w/+; nub-Gal4/Tkv-YFP; Tub-Gal80*^*ts*^*/scrib RNAi*; E: *w; Tkv-YFP/+*; F: *w/+; nub-Gal4/Tkv-YFP; Tub-Gal80*^*ts*^*/+; w/+; nub-Gal4/Tkv-YFP; Tub-Gal80*^*ts*^*/scrib RNAi*

S3A Fig: *w/+; nub-Gal4/+; Tub-Gal80*^*ts*^*/ AP-2α RNAi*; B: *w/+; nub-Gal4/+; Tub-Gal80*^*ts*^*/ AP-2σ RNAi*

S4C Fig: *hsFlp/+; UAS-myr-mRFP/+; tub-Gal4 FRT82B tub-Gal80*/*FRT82B scrib*^*2*^; *hsFlp/+; UAS-myr-mRFP/UAS-Scrib*.*fl-GFP; tub-Gal4 FRT82B tub-Gal80*/*FRT82B scrib*^*2*^; *hsFlp/+; UAS-myr-mRFP/UAS-Scrib*.*LRR*.*LASPD; tub-Gal4 FRT82B tub-Gal80*/*FRT82B scrib*^*2*^; *hsFlp/UAS-myr-deleteLRR; UAS-myr-mRFP/+; tub-Gal4 FRT82B tub-Gal80*/*FRT82B scrib*^*2*^

S6 Fig For *scrib* MARCM: *hsFlp/+; tub-Gal4*, *UAS-mCD8-GFP/+; FRT82B tub-Gal80*/*FRT82B scrib*^*2*^ For *dlg1* MARCM: *FRT19A dlg1*^*B*^*/ hsFlp*, *tub-GAL80*, *FRT19A; tub-GAL4*, *UAS-mCD8-GFP/+*

### Antibodies, chemicals and immunohistochemistry

Pupal wings were fixed in 3.7% formaldehyde (Sigma-Aldrich) at 4°C overnight. Wing imaginal discs were fixed in 3.7% formaldehyde at room temperature (RT) for 20 minutes. All immunostaining and in situ hybridizations were performed as described previously [7,9]. The primary antibodies used are as follows: mouse anti-DLG1, rat anti-DE-Cadherin and mouse anti-GFP (for immunohistochemistry; all at 1:50) were obtained from Developmental Studies Hybridoma Bank, rabbit anti-phospho-SMAD1/5 (1: 200 for IF, 1:2000 for Western blotting) from Cell Signaling Technology (CST), rabbit anti-Rab5 (1:600) and rabbit anti-RFP (1:5000 for Western blotting) from Abcam, mouse anti-RFP (1:5000 for Western blotting) from Chromotek, mouse anti-GFP (1: 5000 for Western blotting) from Millipore, mouse anti-β-tubulin (1:5000) from Sigma-Aldrich, rabbit anti-MYC (1:500), goat anti-Scrib (1:100), rabbit anti-aPKC (1:100) and mouse anti-LGL (1:200) from Santa Cruz Biotechnology, and rabbit anti-Scrib (1:2000) from C. Doe. Secondary antibodies were as follows: goat anti-mouse IgG Alexa 488, goat anti-mouse IgG Alexa 568, goat anti-mouse IgG Alexa 647, goat anti-rabbit IgG Alexa 568, goat anti-rabbit IgG Alexa 647, goat anti-rat IgG Alexa 488 and goat anti-mouse IgG Cy5, all from Molecular Probes (1:200). GFP-booster (1:200, ChromoTek) was used to enhance the YFP signal in Fig 3F and S2C Fig.

### Imaging and image analysis

Fluorescent images were obtained with a Zeiss LSM700 upright confocal microscope. Images of in situ hybridization and adult wings were obtained with a Nikon Eclipse 90i microscope. All confocal immunofluorescent images were processed and analyzed with ImageJ (NIH). The images were the composite of a stack with projection of max intensity unless specified in detailed.

### DNA constructs

Mad-FLAG for cell culture was described previously [44]. Tkv-GFP and caTkv-GFP in UAS plasmids were obtained from G. Marquez. A Punt-GFP construct under UAS control was obtained from M. O’Connor. Full-length (FL) and truncated *scrib* cDNAs were obtained by PCR using *scrib* cDNA (#42064, Addgene) as a template. FL and truncated *scrib* cDNAs with one copy of *MYC* tag at the C-terminus were cloned into BglII and KpnI sites of *pUAST-attB* [45]. Protein sequences of truncated *scrib* cDNAs are as follows: LRR, 1–692; PDZ1/2, 531–1105; PDZ3/4, 928–1400; CT, 1338–1757; LRR+PDZ1/2, 1–1105; ∆CT, 1–1400. *Rab5* cDNAs from the BDGP Gold cDNA collection (*Drosophila* Genomics Resource Center; DGRC) were cloned into *pENTER-D-TOPO* entry vector (Invitrogen) and then subcloned into the destination vector *pTWR* (DGRC). Dominant-negative (S43N) and constitutively-activated (Q88L) forms of Rab5 were generated using an overlap-PCR strategy.

### in vivo RNAi

*nub-GAL4* was used to drive the RNAi in the wing blade in combination with the temperature-sensitive GAL80 [16]. *scrib*, *dlg1* or *lgl* RNAi flies were cultured at 25, 27 or 25°C, respectively. *AP-2* subunit RNAi flies were maintained at 18°C for 5 days and then cultured at 27°C.

### Cell culture and production of recombinant proteins

*Drosophila* S2 cells were used for producing recombinant proteins as previously described [44]. Cells were transfected with HiFugene transfection reagent (Promega) according to the manufacturer’s protocol. S2 cells were transfected with the plasmids expressing indicated cDNA and *tub-GAL4*. Three days after transfection, cells were collected and cell lysates were subjected to immunoprecipitation using the GFP-Nanotrap A Kit (Chromotek) according to the manufacturer's instructions. S2 cells were subjected to the IP lysis buffer (25 mM Tris-HCl pH 7.4, 150 mM NaCl, 1% NP-40, 1 mM EDTA, 5% glycerol) on ice for 30 min. The supernatants obtained were subjected to the WB as an input and subsequent IP. Western blotting was conducted as previously described [44]. All biochemical data shown are representative of no less than three independent assays.

### Reverse transcription-quantitative real-time polymerase chain reaction (RT-qPCR)

Pupal wings were dissected without fixation, and subjected to RNA purification using TRIzol reagent (Invitrogen). RNA template was first converted to cDNA using Maxima First Strand cDNA Synthesis Kit (Thermo Scientific). The cDNA template was then subjected to qPCR carried out with the StepOnePlus Real-Time PCR Systems Kit (Applied Biosystems). The following primers were used:

*gapdh* F, CGAAGATCGGAATTAACGGA;

*gapdh* R, ACCGTGAGTCGAGTCGAATT;

*βTub* F, GAACCCTGCTGATTTCCAAGAT;

*βTub* R, ATATCGTAGAGAGCCTCGTTGT;

*scrib* F, CTGGCATATTCATATCGCACATT;

*scrib* R,TCATCACCTGGCTTCAACA;

*dlg1* F, CACCGAGGATATAACCAGAGAAC;

*dlg1* R, CAGGATGAAGGACACATAGATACC;

*lgl* F, TGAGTCAATCCGCCAACTTCCA;

*lgl* R, TTCACTGTAAGACCAACGCTCTGT;

*vvl* F, CTGCACATACACCATCACAT;

*vvl* R, GGAGAACACATTGCCATAGA.

### Statistics

All experiments were carried out independently at least three times. Error bars indicate s.e.m. Statistical significance was calculated by the two paired *t*-test method.

## Supporting Information

S1 Fig BMP signal regulates *scrib* transcription in the pupal wing. (A) *lgl* RNAi (*nub*^*ts*^ > *lgl* RNAi) adult wing. The PCV position is indicated by an arrow. RNAi flies were cultured at 25°C. (B) Effects of *dlg1* mutant clones on pMad (red) at 24 h AP in the PCV region. *dlg1* mutant cells (green) were generated using MARCM. Dashed box in left panel depicts the region of interest (ROI). Higher magnification pictures of the ROI are shown in the right panels. Nuclei are marked by DAPI (blue) staining. (C-F) GFP:Dpp expressing cells in control (C, E) or *scrib* mutant clones (D, F) were generated using MARCM. Clones were marked by myristoylated RFP (C, D) or GFP (E, F). Note that GFP:Dpp and pMad signal are observed outside the clones when GFP:Dpp are expressed in *scrib* mutant cells. (G, H) Scrib and DE-Cad are up-regulated in the pupal wing by BMP signaling in distinct manner. caTkv clones (labeled by GFP, right panel) were generated using MARCM. Scrib and DE-Cad protein levels were analyzed by anti-Scrib (left) and anti-DE-Cad (middle) antibody staining. Pupal wings were collected at 20 h AP (G) and 24 h AP (H).
(TIF)

S2 Fig Distribution of Tkv is regulated by Scrib. (A) A schematic of different planes (1 and 2) of PCV cells along the apicobasal axis in B. (B) Serial optical sections with 1 μm interval at apical (1) or basal (2) part of PCV cells. Tkv-YFP and Scrib staining in the PCV region at 24 h AP. Loss of Scrib affects Tkv distribution. *scrib* mutant clones are marked by absence of Scrib staining. (C, D) Optical cross sections focused on the PCV region of pupal wing at 24 h AP, showing Tkv-YFP and DLG1 staining in control (*nub*^*ts*^) (C) and *scrib* RNAi (*nub*^*ts*^ *> scrib RNAi*) (D). Note that Tkv is more enriched basally in control, but localizes more apically in *scrib* RNAi wings (arrows). (E) Wild-type pupal wing. Tkv-YFP, Rab5 and DLG1 staining in the PCV region at 24 h AP. (F) Tkv-YFP and Rab5 staining at the basal plane in control (*nub*^*ts*^) and *scrib* RNAi (*nub*^*ts*^ *> scrib RNAi*) in the PCV region at 24 h AP. Arrows indicate that Tkv-YFP puncta co-localize with Rab5. Note that localizations of Tkv and Rab5 in *scrib* RNAi cells are significantly reduced at the basal plane.
(TIF)

S3 Fig AP-2 complex is required for PCV formation. (A, B) *AP-2α* RNAi (*nub*^*ts*^ > *AP-2α* RNAi) (A) and *AP-2σ* RNAi (*nub*^*ts*^ > *AP-2σ* RNAi) adult wings (B). The PCV positions are indicated by arrows.
(TIF)

S4 Fig Interactions of Scrib and Tkv. (A) Co-IP of Scrib and Tkv. Scrib-MYC, Tkv-GFP or caTkv-GFP were expressed in S2 cells, and cell lysates were immunoprecipitated by anti-GFP. Cell lysates (input) and immunoprecipitated proteins (IP: GFP) were analysed by Western blot probed with anti-GFP and anti-MYC antibodies. Note that Scrib-Myc fragments were observed when the blots were analyzed by different conditions (gain: 9) from those in Fig 5B, C (gain: 5) with LiCOR Odyssey. (B) Co-IP of Scrib fragments and LRR domain. MYC-tagged different fragments of Scrib and LRR-RFP were expressed in S2 cells, and cell lysates were immunoprecipitated by anti-RFP. Cell lysates (input) and immunoprecipitated proteins (IP: RFP) were analysed by Western blot probed with anti-RFP and anti-MYC antibodies. Results are representative of one of three independent experiments (A, B). (C) Expression of Scrib (full length), LRR domain or myristoylated PDZ in *scrib* mutant clones labeled by mRFP. DLG1 staining in PCV region at 24 h AP.
(TIF)

S5 Fig Interactions of Scrib, Tkv and Rab5. (A) Co-IP of Rab5 and Tkv. Rab5-RFP and Tkv-GFP or caTkv-GFP were expressed in S2 cells, and cell lysates were immunoprecipitated by anti-GFP. Cell lysates (input) and immunoprecipitated proteins (IP: GFP) were analysed by Western blot probed with anti-GFP and anti-Rab5 antibodies. (B) Wild-type, dominant-negative or constitutively active form of Rab5-RFP and Tkv-GFP were expressed in S2 cells, and cell lysates were immunoprecipitated by anti-GFP. Cell lysates (input) and immunoprecipitated proteins (IP: GFP) were analyzed by Western blot probed with anti-GFP and anti-RFP antibodies. (C) Co-IP of Rab5, Punt and LRR. Rab5-RFP, Punt-GFP and LRR-MYC were expressed in S2 cells, and cell lysates were immunoprecipitated by anti-RFP. Cell lysates (input) and immunoprecipitated proteins (IP: RFP) were analyzed by Western blot probed with anti-GFP, anti-RFP and anti-MYC antibodies. Results are representative of one of three independent experiments (A-C).
(TIF)

S6 Fig Effects of *scrib* or *dlg1* on BMP signaling in the wing imaginal disc. Effects of *scrib* mutant clones (upper panel) or *dlg1* mutant clones (lower panel) on pMad staining (red) in third instar wing imaginal disc. Mutant cell clones were generated using MARCM and labeled with GFP (green). Nuclei are marked by DAPI (blue) staining. Note that pMad signal appears to be normal in *scrib* or *dlg1* mutant cells in the wing imaginal disc.
(TIF)

## References

[1] Rodriguez-Boulan E, Macara IG (2014) Organization and execution of the epithelial polarity programme. Nat Rev Mol Cell Biol 15: 225–242. 10.1038/nrm3775 24651541 PMC4211427

[2] Martin-Belmonte F, Perez-Moreno M (2012) Epithelial cell polarity, stem cells and cancer. Nat Rev Cancer 12: 23–38. 10.1038/nrc3169 22169974

[3] Bilder D (2004) Epithelial polarity and proliferation control: links from the Drosophila neoplastic tumor suppressors. Genes Dev 18: 1909–1925. 10.1101/gad.1211604 15314019

[4] Bilder D, Perrimon N (2000) Localization of apical epithelial determinants by the basolateral PDZ protein Scribble. Nature 403: 676–680. 10.1038/35001108 10688207

[5] Bilder D, Li M, Perrimon N (2000) Cooperative regulation of cell polarity and growth by Drosophila tumor suppressors. Science 289: 113–116. 10884224 10.1126/science.289.5476.113

[6] Shimmi O, Newfeld SJ (2013) New insights into extracellular and post-translational regulation of TGF-beta family signalling pathways. J Biochem 154: 11–19. 10.1093/jb/mvt046 23698094 PMC3693483

[7] Matsuda S, Shimmi O (2012) Directional transport and active retention of Dpp/BMP create wing vein patterns in Drosophila. Dev Biol 366: 153–162. 10.1016/j.ydbio.2012.04.009 22542596

[8] Ralston A, Blair SS (2005) Long-range Dpp signaling is regulated to restrict BMP signaling to a crossvein competent zone. Dev Biol 280: 187–200. 10.1016/j.ydbio.2005.01.018 15766758

[9] Matsuda S, Blanco J, Shimmi O (2013) A feed-forward loop coupling extracellular BMP transport and morphogenesis in Drosophila wing. PLoS Genet 9: e1003403 10.1371/journal.pgen.1003403 23555308 PMC3605110

[10] Blair SS (2007) Wing vein patterning in Drosophila and the analysis of intercellular signaling. Annu Rev Cell Dev Biol 23: 293–319. 10.1146/annurev.cellbio.23.090506.123606 17506700

[11] Shimmi O, Ralston A, Blair SS, O'Connor MB (2005) The crossveinless gene encodes a new member of the Twisted gastrulation family of BMP-binding proteins which, with Short gastrulation, promotes BMP signaling in the crossveins of the Drosophila wing. Dev Biol 282: 70–83. 10.1016/j.ydbio.2005.02.029 15936330

[12] Conley CA, Silburn R, Singer MA, Ralston A, Rohwer-Nutter D, et al (2000) Crossveinless 2 contains cysteine-rich domains and is required for high levels of BMP-like activity during the formation of the cross veins in Drosophila. Development 127: 3947–3959. 10952893 10.1242/dev.127.18.3947

[13] Chen J, Honeyager SM, Schleede J, Avanesov A, Laughon A, et al (2012) Crossveinless d is a vitellogenin-like lipoprotein that binds BMPs and HSPGs, and is required for normal BMP signaling in the Drosophila wing. Development 139: 2170–2176. 10.1242/dev.073817 22573617 PMC3357910

[14] Christoforou CP, Greer CE, Challoner BR, Charizanos D, Ray RP (2008) The detached locus encodes Drosophila Dystrophin, which acts with other components of the Dystrophin Associated Protein Complex to influence intercellular signalling in developing wing veins. Dev Biol 313: 519–532. 10.1016/j.ydbio.2007.09.044 18093579

[15] Denholm B, Brown S, Ray RP, Ruiz-Gomez M, Skaer H, et al (2005) crossveinless-c is a RhoGAP required for actin reorganisation during morphogenesis. Development 132: 2389–2400. 10.1242/dev.01829 15843408

[16] McGuire SE, Le PT, Osborn AJ, Matsumoto K, Davis RL (2003) Spatiotemporal rescue of memory dysfunction in Drosophila. Science 302: 1765–1768. 10.1126/science.1089035 14657498

[17] Tanimoto H, Itoh S, ten Dijke P, Tabata T (2000) Hedgehog creates a gradient of DPP activity in Drosophila wing imaginal discs. Mol Cell 5: 59–71. 10678169 10.1016/s1097-2765(00)80403-7

[18] Kang JS, Liu C, Derynck R (2009) New regulatory mechanisms of TGF-beta receptor function. Trends Cell Biol 19: 385–394. 10.1016/j.tcb.2009.05.008 19648010

[19] de Vreede G, Schoenfeld JD, Windler SL, Morrison H, Lu H, et al (2014) The Scribble module regulates retromer-dependent endocytic trafficking during epithelial polarization. Development 141: 2796–2802. 10.1242/dev.105403 25005475 PMC4197622

[20] McMahon HT, Boucrot E (2011) Molecular mechanism and physiological functions of clathrin-mediated endocytosis. Nat Rev Mol Cell Biol 12: 517–533. 10.1038/nrm3151 21779028

[21] Evans TA, Haridas H, Duffy JB (2009) Kekkon5 is an extracellular regulator of BMP signaling. Dev Biol 326: 36–46. 10.1016/j.ydbio.2008.10.002 19013143

[22] Zeitler J, Hsu CP, Dionne H, Bilder D (2004) Domains controlling cell polarity and proliferation in the Drosophila tumor suppressor Scribble. J Cell Biol 167: 1137–1146. 10.1083/jcb.200407158 15611336 PMC2172630

[23] Entchev EV, Schwabedissen A, Gonzalez-Gaitan M (2000) Gradient formation of the TGF-beta homolog Dpp. Cell 103: 981–991. 11136982 10.1016/s0092-8674(00)00200-2

[24] de Celis JF (1997) Expression and function of decapentaplegic and thick veins during the differentiation of the veins in the Drosophila wing. Development 124: 1007–1018. 9056776 10.1242/dev.124.5.1007

[25] Lecuit T, Cohen SM (1998) Dpp receptor levels contribute to shaping the Dpp morphogen gradient in the Drosophila wing imaginal disc. Development 125: 4901–4907. 9811574 10.1242/dev.125.24.4901

[26] Funakoshi Y, Minami M, Tabata T (2001) mtv shapes the activity gradient of the Dpp morphogen through regulation of thickveins. Development 128: 67–74. 11092812 10.1242/dev.128.1.67

[27] Serpe M, Umulis D, Ralston A, Chen J, Olson DJ, et al (2008) The BMP-binding protein Crossveinless 2 is a short-range, concentration-dependent, biphasic modulator of BMP signaling in Drosophila. Dev Cell 14: 940–953. 10.1016/j.devcel.2008.03.023 18539121 PMC2488203

[28] Piguel NH, Fievre S, Blanc JM, Carta M, Moreau MM, et al (2014) Scribble1/AP2 complex coordinates NMDA receptor endocytic recycling. Cell Rep 9: 712–727. 10.1016/j.celrep.2014.09.017 25310985

[29] Chen CL, Schroeder MC, Kango-Singh M, Tao C, Halder G (2012) Tumor suppression by cell competition through regulation of the Hippo pathway. Proc Natl Acad Sci U S A 109: 484–489. 10.1073/pnas.1113882109 22190496 PMC3258595

[30] Moreno E, Basler K, Morata G (2002) Cells compete for decapentaplegic survival factor to prevent apoptosis in Drosophila wing development. Nature 416: 755–759. 10.1038/416755a 11961558

[31] Aoyama M, Sun-Wada GH, Yamamoto A, Yamamoto M, Hamada H, et al (2012) Spatial restriction of bone morphogenetic protein signaling in mouse gastrula through the mVam2-dependent endocytic pathway. Dev Cell 22: 1163–1175. 10.1016/j.devcel.2012.05.009 22698281

[32] Kim S, Wairkar YP, Daniels RW, DiAntonio A (2010) The novel endosomal membrane protein Ema interacts with the class C Vps-HOPS complex to promote endosomal maturation. J Cell Biol 188: 717–734. 10.1083/jcb.200911126 20194640 PMC2835942

[33] Liu Z, Huang Y, Hu W, Huang S, Wang Q, et al (2014) dAcsl, the Drosophila ortholog of acyl-CoA synthetase long-chain family member 3 and 4, inhibits synapse growth by attenuating bone morphogenetic protein signaling via endocytic recycling. J Neurosci 34: 2785–2796. 10.1523/JNEUROSCI.3547-13.2014 24553921 PMC6608520

[34] O'Connor-Giles KM, Ho LL, Ganetzky B (2008) Nervous wreck interacts with thickveins and the endocytic machinery to attenuate retrograde BMP signaling during synaptic growth. Neuron 58: 507–518. 10.1016/j.neuron.2008.03.007 18498733 PMC2448395

[35] Wang X, Shaw WR, Tsang HT, Reid E, O'Kane CJ (2007) Drosophila spichthyin inhibits BMP signaling and regulates synaptic growth and axonal microtubules. Nat Neurosci 10: 177–185. 10.1038/nn1841 17220882 PMC2464677

[36] Umasankar PK, Sanker S, Thieman JR, Chakraborty S, Wendland B, et al (2012) Distinct and separable activities of the endocytic clathrin-coat components Fcho1/2 and AP-2 in developmental patterning. Nat Cell Biol 14: 488–501. 10.1038/ncb2473 22484487 PMC3354769

[37] Gleason RJ, Akintobi AM, Grant BD, Padgett RW (2014) BMP signaling requires retromer-dependent recycling of the type I receptor. Proc Natl Acad Sci U S A 111: 2578–2583. 10.1073/pnas.1319947111 24550286 PMC3932876

[38] Morawa KS, Schneider M, Klein T (2015) Lgd regulates the activity of the BMP/Dpp signalling pathway during Drosophila oogenesis. Development 142: 1325–1335. 10.1242/dev.112961 25804739

[39] Belenkaya TY, Han C, Yan D, Opoka RJ, Khodoun M, et al (2004) Drosophila Dpp morphogen movement is independent of dynamin-mediated endocytosis but regulated by the glypican members of heparan sulfate proteoglycans. Cell 119: 231–244. 10.1016/j.cell.2004.09.031 15479640

[40] Lighthouse DV, Buszczak M, Spradling AC (2008) New components of the Drosophila fusome suggest it plays novel roles in signaling and transport. Dev Biol 317: 59–71. 10.1016/j.ydbio.2008.02.009 18355804 PMC2410214

[41] Morin X, Daneman R, Zavortink M, Chia W (2001) A protein trap strategy to detect GFP-tagged proteins expressed from their endogenous loci in Drosophila. Proc Natl Acad Sci U S A 98: 15050–15055. 10.1073/pnas.261408198 11742088 PMC64981

[42] Gonzalez-Gaitan M, Jackle H (1999) The range of spalt-activating Dpp signalling is reduced in endocytosis-defective Drosophila wing discs. Mech Dev 87: 143–151. 10495278 10.1016/s0925-4773(99)00156-2

[43] Lee T, Luo L (1999) Mosaic analysis with a repressible cell marker for studies of gene function in neuronal morphogenesis. Neuron 22: 451–461. 10197526 10.1016/s0896-6273(00)80701-1

[44] Kunnapuu J, Tauscher PM, Tiusanen N, Nguyen M, Loytynoja A, et al (2014) Cleavage of the Drosophila screw prodomain is critical for a dynamic BMP morphogen gradient in embryogenesis. Dev Biol 389: 149–159. 10.1016/j.ydbio.2014.02.007 24560644

[45] Bischof J, Maeda RK, Hediger M, Karch F, Basler K (2007) An optimized transgenesis system for Drosophila using germ-line-specific phiC31 integrases. Proc Natl Acad Sci U S A 104: 3312–3317. 10.1073/pnas.0611511104 17360644 PMC1805588

