# Supplementary figures and images for: Scribbled Optimizes BMP Signaling through Its Receptor Internalization to the Rab5 Endosome and Promote Robust Epithelial Morphogenesis
Source: PLoS Genet. 2016 Nov 4;12(11):e1006424. doi: 10.1371/journal.pgen.1006424 (PMC5096713; doi:10.1371/journal.pgen.1006424)

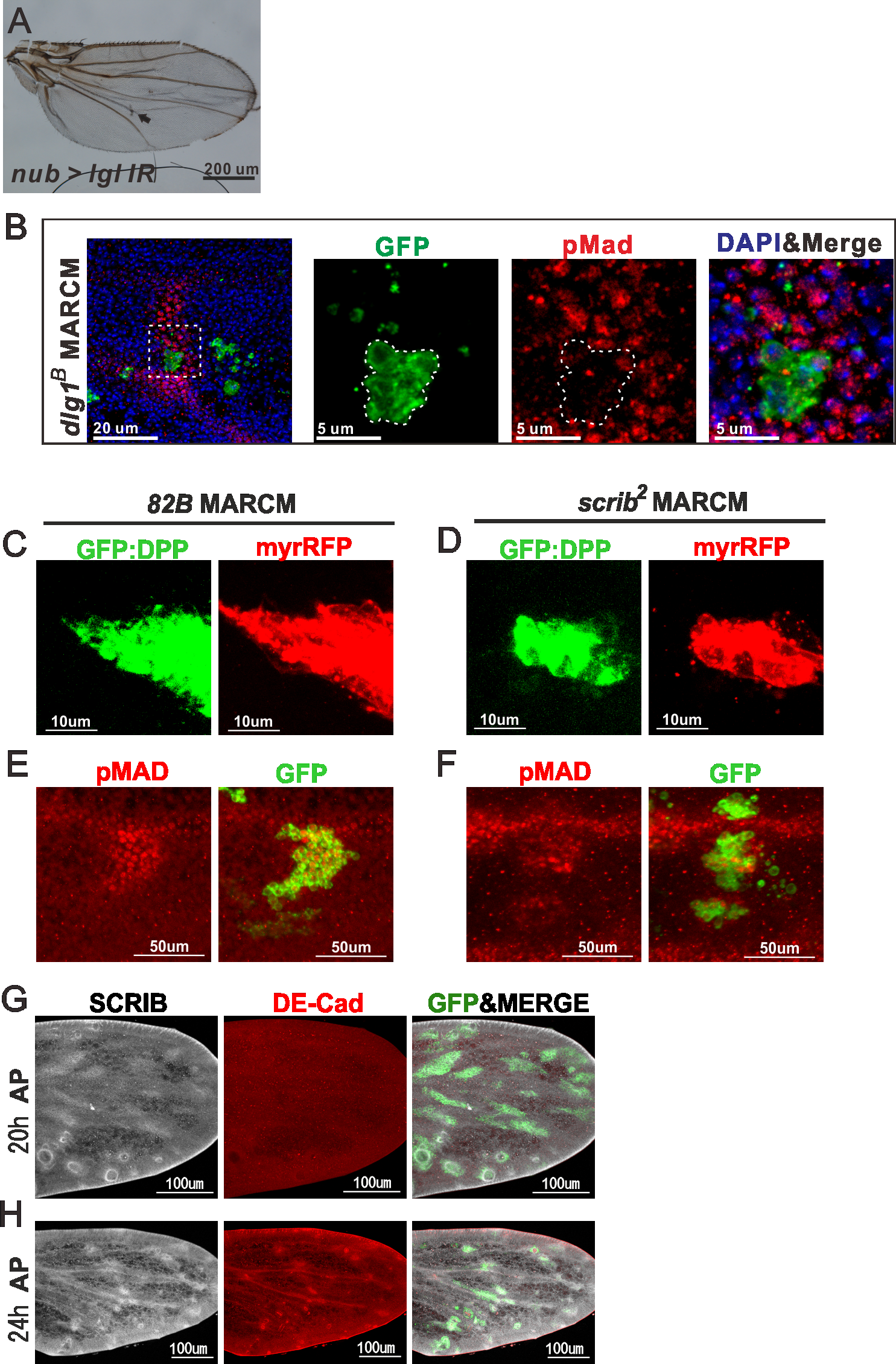

Supplement: S1 Fig — (A) lgl RNAi (nubts > lgl RNAi) adult wing. The PCV position is indicated by an arrow. RNAi flies were cultured at 25°C. (B) Effects of dlg1 mutant clones on pMad (red) at 24 h AP in the PCV region. dlg1 mutant cells (green) were generated using MARCM. Dashed box in left panel depicts the region of interest (ROI). Higher magnification pictures of the ROI are shown in the right panels. Nuclei are marked by DAPI (blue) staining. (C-F) GFP:Dpp expressing cells in control (C, E) or scrib mutant clones (D, F) were generated using MARCM. Clones were marked by myristoylated RFP (C, D) or GFP (E, F). Note that GFP:Dpp and pMad signal are observed outside the clones when GFP:Dpp are expressed in scrib mutant cells. (G, H) Scrib and DE-Cad are up-regulated in the pupal wing by BMP signaling in distinct manner. caTkv clones (labeled by GFP, right panel) were generated using MARCM. Scrib and DE-Cad protein levels were analyzed by anti-Scrib (left) and anti-DE-Cad (middle) antibody staining. Pupal wings were collected at 20 h AP (G) and 24 h AP (H). (TIF) [file pgen.1006424.s001.tif]

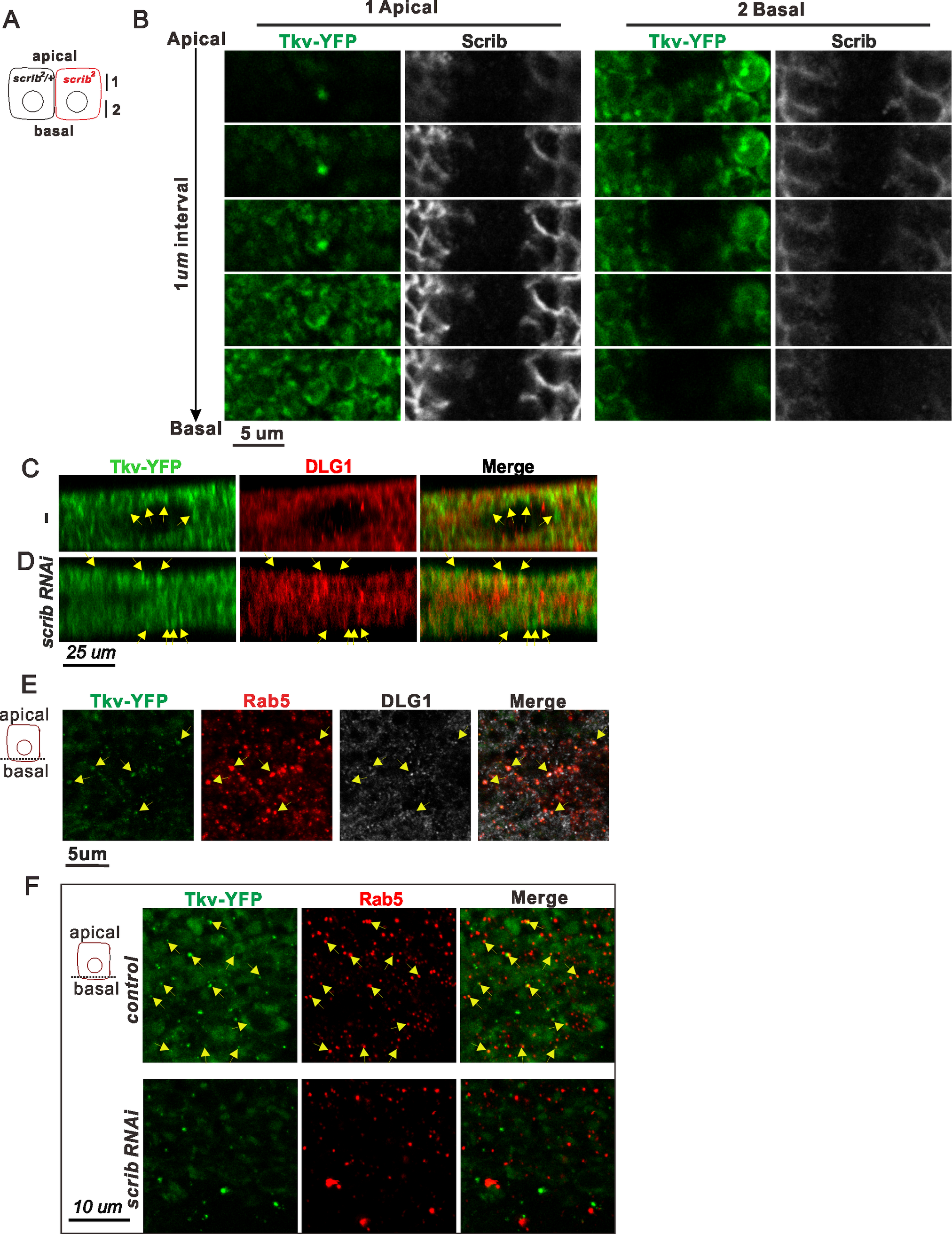

Supplement: S2 Fig — (A) A schematic of different planes (1 and 2) of PCV cells along the apicobasal axis in B. (B) Serial optical sections with 1 μm interval at apical (1) or basal (2) part of PCV cells. Tkv-YFP and Scrib staining in the PCV region at 24 h AP. Loss of Scrib affects Tkv distribution. scrib mutant clones are marked by absence of Scrib staining. (C, D) Optical cross sections focused on the PCV region of pupal wing at 24 h AP, showing Tkv-YFP and DLG1 staining in control (nubts) (C) and scrib RNAi (nubts > scrib RNAi) (D). Note that Tkv is more enriched basally in control, but localizes more apically in scrib RNAi wings (arrows). (E) Wild-type pupal wing. Tkv-YFP, Rab5 and DLG1 staining in the PCV region at 24 h AP. (F) Tkv-YFP and Rab5 staining at the basal plane in control (nubts) and scrib RNAi (nubts > scrib RNAi) in the PCV region at 24 h AP. Arrows indicate that Tkv-YFP puncta co-localize with Rab5. Note that localizations of Tkv and Rab5 in scrib RNAi cells are significantly reduced at the basal plane. (TIF) [file pgen.1006424.s002.tif]

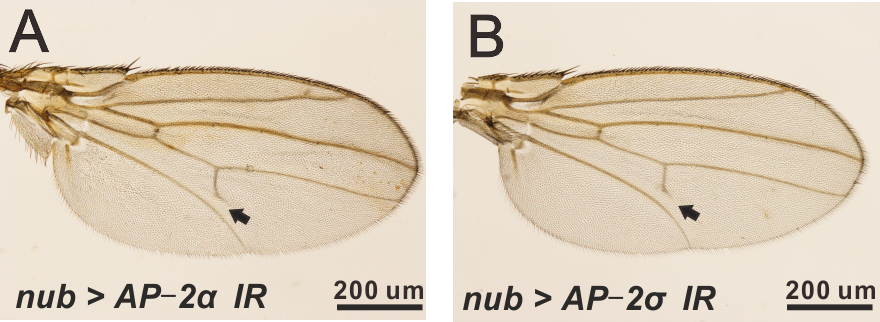

Supplement: S3 Fig — (A, B) AP-2α RNAi (nubts > AP-2α RNAi) (A) and AP-2σ RNAi (nubts > AP-2σ RNAi) adult wings (B). The PCV positions are indicated by arrows. (TIF) [file pgen.1006424.s003.tif]

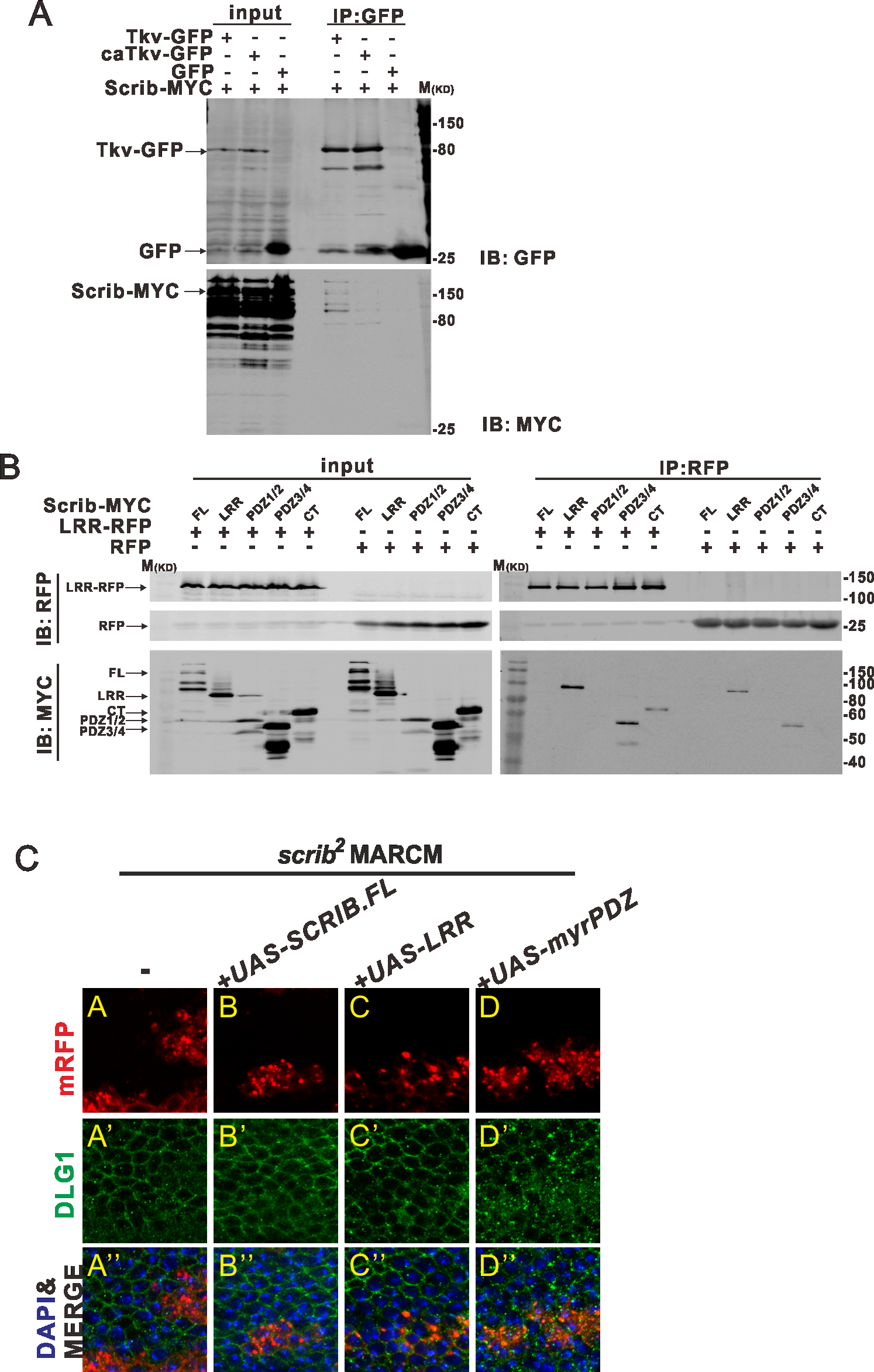

Supplement: S4 Fig — (A) Co-IP of Scrib and Tkv. Scrib-MYC, Tkv-GFP or caTkv-GFP were expressed in S2 cells, and cell lysates were immunoprecipitated by anti-GFP. Cell lysates (input) and immunoprecipitated proteins (IP: GFP) were analysed by Western blot probed with anti-GFP and anti-MYC antibodies. Note that Scrib-Myc fragments were observed when the blots were analyzed by different conditions (gain: 9) from those in Fig 5B, C (gain: 5) with LiCOR Odyssey. (B) Co-IP of Scrib fragments and LRR domain. MYC-tagged different fragments of Scrib and LRR-RFP were expressed in S2 cells, and cell lysates were immunoprecipitated by anti-RFP. Cell lysates (input) and immunoprecipitated proteins (IP: RFP) were analysed by Western blot probed with anti-RFP and anti-MYC antibodies. Results are representative of one of three independent experiments (A, B). (C) Expression of Scrib (full length), LRR domain or myristoylated PDZ in scrib mutant clones labeled by mRFP. DLG1 staining in PCV region at 24 h AP. (TIF) [file pgen.1006424.s004.tif]

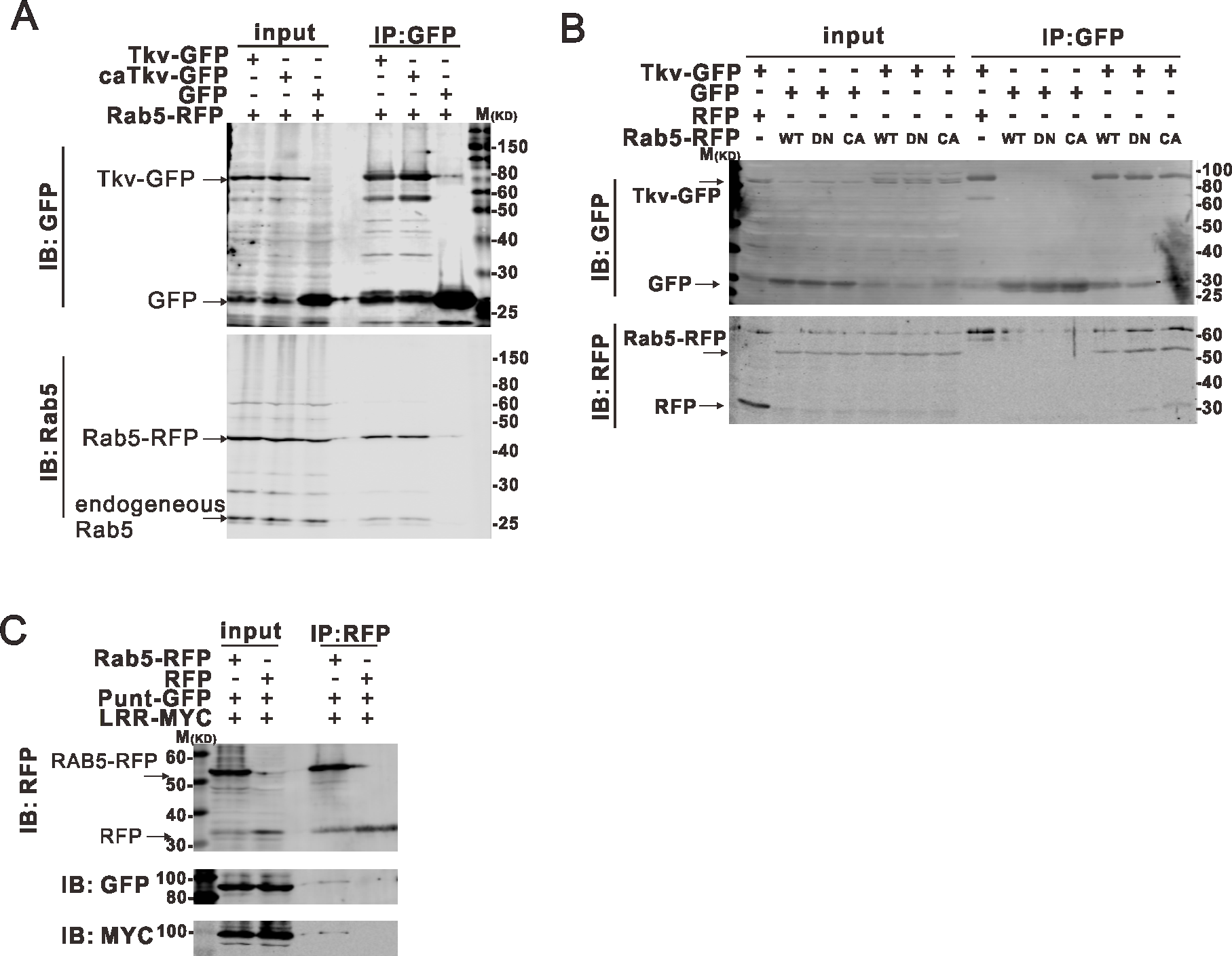

Supplement: S5 Fig — (A) Co-IP of Rab5 and Tkv. Rab5-RFP and Tkv-GFP or caTkv-GFP were expressed in S2 cells, and cell lysates were immunoprecipitated by anti-GFP. Cell lysates (input) and immunoprecipitated proteins (IP: GFP) were analysed by Western blot probed with anti-GFP and anti-Rab5 antibodies. (B) Wild-type, dominant-negative or constitutively active form of Rab5-RFP and Tkv-GFP were expressed in S2 cells, and cell lysates were immunoprecipitated by anti-GFP. Cell lysates (input) and immunoprecipitated proteins (IP: GFP) were analyzed by Western blot probed with anti-GFP and anti-RFP antibodies. (C) Co-IP of Rab5, Punt and LRR. Rab5-RFP, Punt-GFP and LRR-MYC were expressed in S2 cells, and cell lysates were immunoprecipitated by anti-RFP. Cell lysates (input) and immunoprecipitated proteins (IP: RFP) were analyzed by Western blot probed with anti-GFP, anti-RFP and anti-MYC antibodies. Results are representative of one of three independent experiments (A-C). (TIF) [file pgen.1006424.s005.tif]

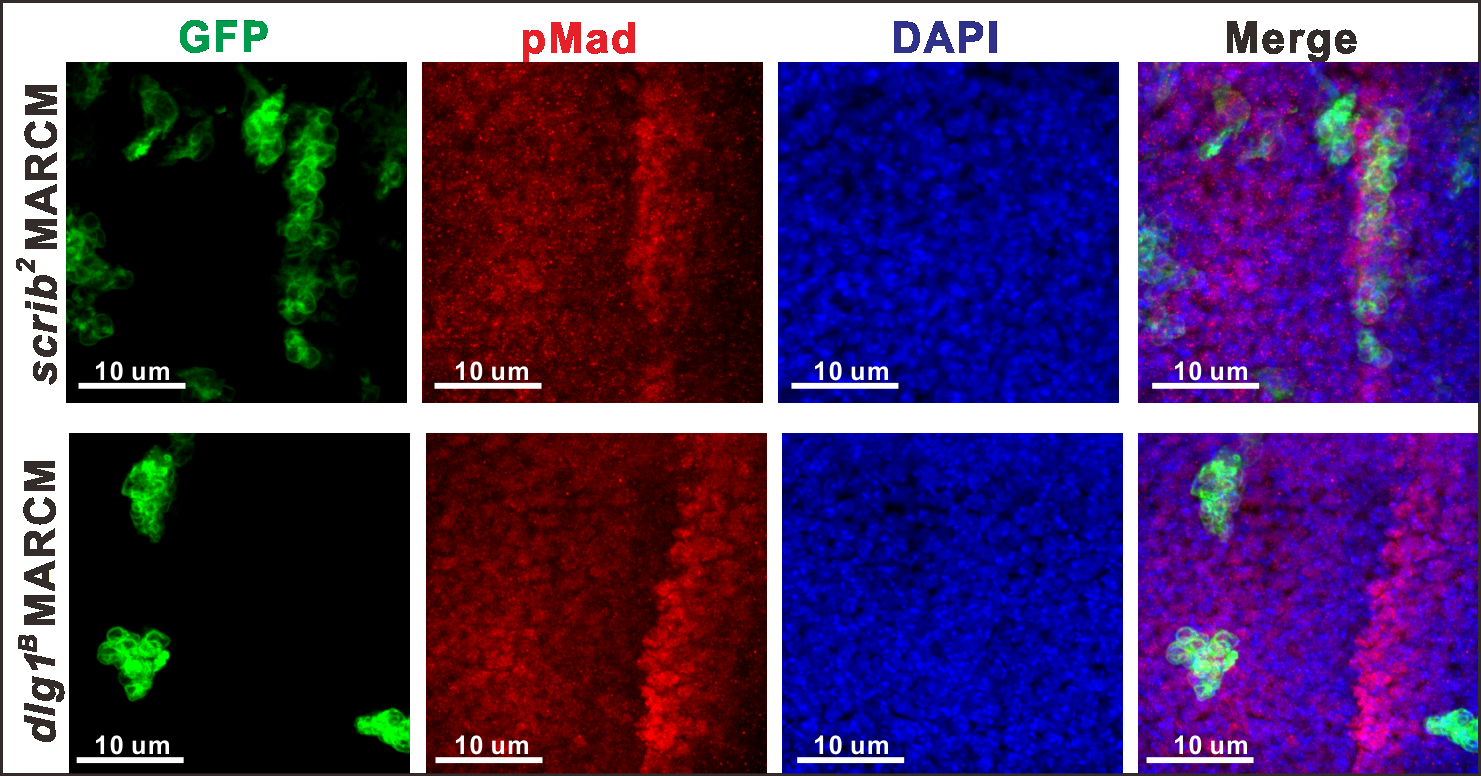

Supplement: S6 Fig — Effects of scrib mutant clones (upper panel) or dlg1 mutant clones (lower panel) on pMad staining (red) in third instar wing imaginal disc. Mutant cell clones were generated using MARCM and labeled with GFP (green). Nuclei are marked by DAPI (blue) staining. Note that pMad signal appears to be normal in scrib or dlg1 mutant cells in the wing imaginal disc. (TIF) [file pgen.1006424.s006.tif]
